# Reference-State Error Mitigation: A Strategy for High Accuracy Quantum Computation of Chemistry

**DOI:** 10.1021/acs.jctc.2c00807

**Published:** 2023-01-27

**Authors:** Phalgun Lolur, Mårten Skogh, Werner Dobrautz, Christopher Warren, Janka Biznárová, Amr Osman, Giovanna Tancredi, Göran Wendin, Jonas Bylander, Martin Rahm

**Affiliations:** † Department of Chemistry and Chemical Engineering, Chalmers University of Technology, SE-412 96 Gothenburg, Sweden; ‡ Data Science & Modelling, Pharmaceutical Science, R&D, AstraZeneca, SE-431 83 Mölndal, Gothenburg, Sweden; § Department of Microtechnology and Nanoscience MC2, Chalmers University of Technology, SE-412 96 Gothenburg, Sweden

## Abstract

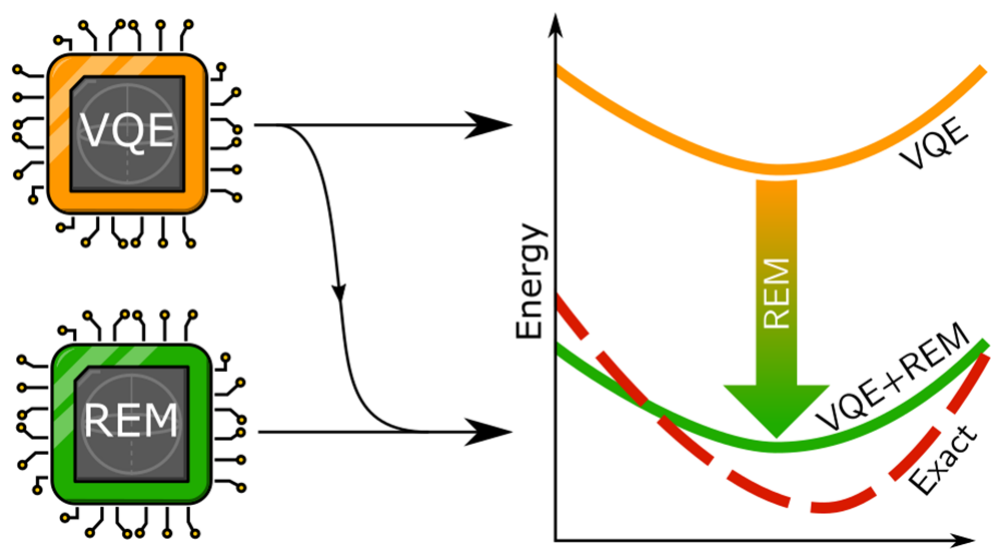

Decoherence and gate errors severely limit the capabilities of state-of-the-art quantum computers. This work introduces a strategy for reference-state error mitigation (REM) of quantum chemistry that can be straightforwardly implemented on current and near-term devices. REM can be applied alongside existing mitigation procedures, while requiring minimal postprocessing and only one or no additional measurements. The approach is agnostic to the underlying quantum mechanical ansatz and is designed for the variational quantum eigensolver. Up to two orders-of-magnitude improvement in the computational accuracy of ground state energies of small molecules (H_2_, HeH^+^, and LiH) is demonstrated on superconducting quantum hardware. Simulations of noisy circuits with a depth exceeding 1000 two-qubit gates are used to demonstrate the scalability of the method.

## Introduction

Quantum computers hold a potential for solving problems that are intractable on current and future computers.^1,2^ Quantum chemistry is one of the research areas where *quantum advantage* is expected in the near future.^3−6^ One of the major challenges in realizing practical quantum computation of chemistry is the sensitivity of quantum devices to noise. Errors due to noise can be caused by several factors such as spontaneous emission, control and measurement imperfection, and unwanted coupling with the environment.^7^ Whereas reliable error correction is expected in future quantum computers, such *fault-tolerant* machines will put high demands on both quality and number of physical qubits.^4^ Increasingly robust hybrid algorithms^2,8−10^ are being designed for quantum chemistry on near-term, noisy intermediate-scale quantum (NISQ) devices.^11^ Unfortunately, noise causes such algorithms to produce results, such as energies of molecules, that are of relatively low quality, even as they rely on shallow quantum circuits.^12−15^ We will return to discuss how one can define quality in terms of accuracy and precision in this context.

The general challenge of noise in quantum hardware has motivated the development of several methods for *error mitigation*:^16^ readout/measurement error mitigation,^17^ zero noise/Richardson extrapolation,^18,19^ Clifford data regression,^20^ training by fermionic linear optics (TFLO),^21^ rescaling as per Arute et al.,^22^ probabilistic error cancellation,^23^ quantum subspace expansion,^24^ postselection,^25^ McWeeny purification,^26^ virtual state distillation,^27^ and symmetry verification^28^ are some examples of techniques exploited to improve the quality of measurements of encoded Hamiltonians through pre- or postprocessing. Some of these techniques have been shown to offer improvements when computing energies of small molecules with variational algorithms.^26,29^ A combination of mitigation strategies is often a good approach to minimize errors.

In this study, we report on a chemistry-inspired error-mitigation strategy that can be combined with any variant of the variational quantum eigensolver^9,30^ (VQE). Our approach, reference-state error mitigation (REM), relies on postprocessing that can be readily performed on a classical computer. The method is applicable across a wide range of noise intensities and is low-cost in that it requires an overhead of at most one additional VQE energy evaluation. REM can readily be employed together with other error mitigation methods, and throughout this work we additionally use readout mitigation, which corrects for hardware-specific nonideal correlation between prepared and measured states.^17,22^ Readout mitigation works by performing an initial calibration against a subset of Clifford gates, whereby known states are prepared and measured; see the Supporting Information (SI).

The evaluation of *computational accuracy* in this work should not be confused with *chemical accuracy.*(31) We here use the term *computational accuracy* specifically when comparing results of a quantum calculation with the exact solution *at that same level of theory*. Computational accuracy then, in the context of VQE calculations, refers to how accurate a given VQE problem is solved with respect to the given Hamiltonian and ansatz. This accuracy can only be quantified so long as it is possible to solve the problem without noise, e.g., using conventional quantum chemistry (which we can still do; discussing the limits of conventional quantum chemistry methods is outside the scope of this work). Practical implementations of VQE on real hardware currently suffer from drastic deficiencies in level of theory, basis set size,^32^ proper consideration of the physical environment (e.g., solvent effects), and dynamical effects. These limitations keep quantum computation (including our own) from accurately predicting real chemical processes. On the other hand, *chemical accuracy* is the correct term for what is required to make realistic predictions and is commonly defined as an error of 1 kcal/mol (∼1.6 millihartree) from the exact solution.^9,15,26,33^ We encourage the community to use the appropriate terminology. The hunt for chemical accuracy in the NISQ era is far from over.

## Methods

**Figure 1:**
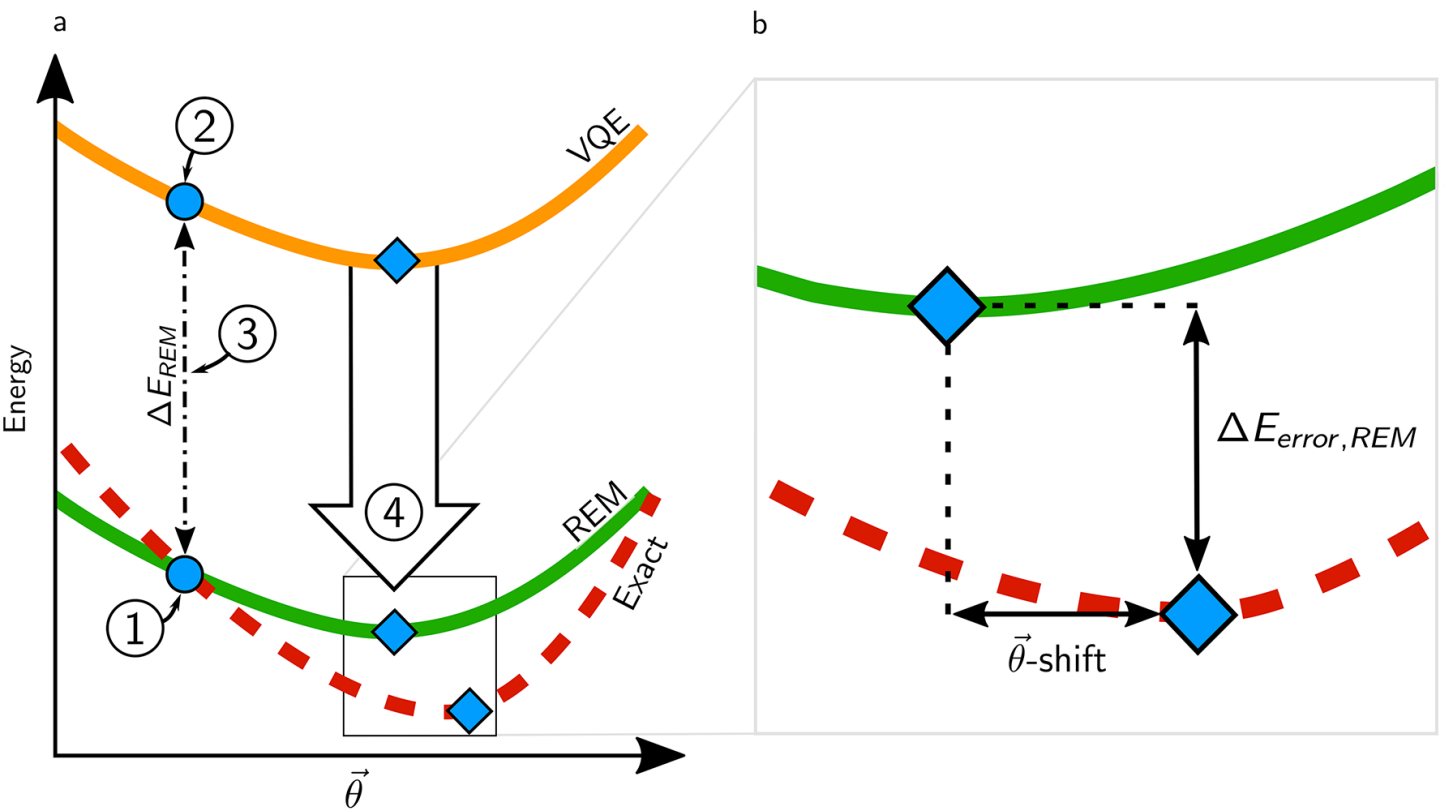
(a) One-dimensional representation of the electronic energy *E* as a function of quantum circuit parameters *θ⃗*. The REM approach can be explained as a four-step process: (1) A computationally tractable reference solution (such as Hartree–Fock) is computed on a classical computer. (2) A quantum measurement of the VQE surface (orange) is made using parameters corresponding to the reference solution. (3) The difference in energy calculated for the reference solution on classical and quantum hardware defines an error, Δ*E*_*REM*_. (4) The error estimate is assumed to be systematic and is used to correct the VQE surface in the proximity of the reference coordinate. The resulting REM corrected surface and the exact (noise-free) solution are represented by solid green and dashed red lines, respectively. Blue dots represent the coordinates of the reference calculations, while diamonds indicate minima of the different energy landscapes. (b) The inset shows the remaining error after application of REM. A possible difference in the location of the minima is indicated by *θ⃗*-shift (b). The difference in energy between the VQE and REM minima is indicated by Δ*E*_*error*,*REM*_.

The goal of the VQE algorithm is to minimize the electronic energy with respect to a set of quantum circuit parameters, i.e.,
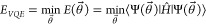
1where *θ⃗* = [θ_1_, θ_2_, ..., *θ*_*n*_], *Ĥ* is the molecular Hamiltonian, and |Ψ(*θ⃗*)⟩ represents the parametrized trial state generated by the VQE circuit. The VQE energy, *E*_*VQE*_(*θ⃗*), can therefore be thought of as living on an *n*-dimensional surface in parameter space. A one-dimensional representation of such a surface is shown in orange at the top of Figure 1. The *E*_*VQE*_(*θ⃗*) surface is associated with some degree of systematic and random noise that can only be partially removed by accounting for state-preparation and measurement errors through readout mitigation.

As the name suggests, the REM method rests on an appropriate choice of a reference wave function, or reference state. We recommend the reference state is (a) chemically motivated, i.e., likely physically similar to the sought state, and (b) fast (or at least viable) to evaluate using a classical computer. These properties are often also desired in the choice of initial guess for VQE, thus the initial point for optimization is commonly a good choice of reference state. The Hartree–Fock state is a practical example of an often-suitable reference wave function that is based on a computationally efficient mean-field description of the electronic potential. We rely on Hartree–Fock as the reference state in this work, but we will investigate different reference choices, better suited to more strongly correlated problems, in future work.

For the REM method to be practically useful, the cost associated with calculating the reference state has to be *lower* than the actual VQE calculation. One way to evaluate the relative cost of classical as compared to quantum computation is to compare their respective computational complexity. A lower bound estimate of the cost of VQE is the number of measurements required to evaluate the energy. In an ideal scenario, i.e., without noise, such measurements approximately scale as *O*(*n*^4^), where *n* is the number of basis functions. In contrast, the cost of conventional Hartree–Fock calculations have a practical scaling between *O*(*n*^2^) and *O*(*n*^4^).^34^

Once the parametrized reference state |Ψ(*θ⃗*_*ref*_)⟩ is prepared, a determination of the resulting energy error Δ*E*_*REM*_ at the reference parameters can be made,
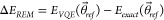
2where *E*_*exact*_(*θ⃗*_*ref*_) is the exact solution (up to numerical precision) for the reference state, evaluated on a classical computer. *E*_*VQE*_(*θ⃗*_*ref*_) refers to the energy evaluated from measurements on a quantum computer at the reference parameter value, *θ⃗*_*ref*_. The idea of approaching error mitigation by comparing noisy measurements to noise-free tractable classical calculations shares some commonality with mitigation techniques such as Clifford data regression, TFLO, and the rescaling technique of Arute et al.^22^ However, our method is distinct from these methods in that REM only relies on the use of *a single* conventional calculation to generate a reference state. Because REM incorporates mitigation through the choice of a chemically and physically motivated initial guess for the VQE algorithm it does not require training on a large number of measured expectation values, as Clifford data regression and TFLO do. REM also considers the effects of both circuit depth and composition, which are often overlooked by other methods.

It is important to make the distinction between the initial state in the VQE calculation and the reference state, which need not be the same. The reference state can either be a part of the VQE optimization or be prepared and measured separately from the variational procedure. Provided that the reference state is also used as an initial guess for the VQE algorithm, it is possible to perform REM without incurring *any* additional measurement cost.

The exact energy at any arbitrary coordinate, *E*_*exact*_(*θ⃗*), can be expressed as
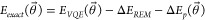
3where Δ*E*_*p*_(*θ⃗*) includes any parameter-dependence of noise present and Δ*E*_*p*_(*θ⃗*_*ref*_) = 0. The underlying assumption of the REM method is that such parameter dependence of the noise is negligible close to the reference geometry, i.e.,  where Δ*θ⃗* = | *θ⃗* – *θ⃗*_*ref*_| . In other words, the effectiveness of the REM approach can be assumed dependent on the Euclidean distance of the reference state to the exact solution |*θ⃗*_*exact*_ – *θ⃗*_*ref*_|, given that both are in the same convex region of the energy surface. When this approximation fails, noise can shift features in the energy surface, such as the optimal coordinates identified using the VQE algorithm, *θ⃗*_*min*,*VQE*_, away from the true minimum, *θ⃗*_*min*,*exact*_(Figure 1b), resulting in a *θ⃗*-shift. When evaluating our method, we will not quantify Δ*E*_*p*_(*θ⃗*) but instead compare energies obtained for the two minima on the exact and the VQE surface,
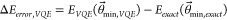
4In eq 4, *E*_*exact*_(*θ⃗*_*min*,*exact*_) is the exact solution obtained by an ideal noise-free VQE optimization and *E*_*VQE*_(*θ⃗*_*min*,*VQE*_) is the energy of a converged noisy VQE optimization. The error remaining after applying REM to a converged noisy VQE optimization is
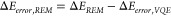
5

## Results and Discussion

To assess the reliability of REM, we have implemented it for the ground state energy computation of small molecules on two current NISQ devices, the ibmq_quito of IBMQ and the Särimner device of Chalmers University. Details of hardware, circuits, measurements and estimates on confidence bounds are provided in the SI. Table 1 shows an amalgamation of our measurement results for the ground state energy of the hydrogen molecule (H_2_), helium hydride (HeH^+^), and lithium hydride (LiH). The ansatze^3^ used for the H_2_ and HeH^+^ molecules are chemistry-inspired and based on unitary coupled cluster theory,^35^ whereas a hardware-efficient ansatz is used for LiH. Table 1 also includes results of simulations of LiH and beryllium hydride (BeH_2_). The latter circuits are substantially larger than what is feasible on current devices as they would incur insurmountable errors due to noise. Combined, this test set ranges from a two-qubit circuit with just 1 two-qubit gate for H_2_, to a six-qubit circuit with 1096 two-qubit gates for BeH_2_ (Table 2). Our simulations of BeH_2_ contains 26 variational parameters (Table 2).

**Table 1:** Total Ground State Energies of Molecules at Experimental Equilibrium Distances^a^, without and with the Application of REM^b^

Molecule^a^	*E*_*exact*_(*θ⃗*_*min*_)	*E*_*VQE*_(*θ⃗*_*min*,*VQE*_)	*E*_*REM*_	Δ*E*_*error*,*VQE*_	Δ*E*_*error*,*REM*_
H_2_^c^	–1.1373	–1.1085(60)	–1.1355	0.029(6)	0.002(8)
HeH^+^^d^	–2.8542	–2.825(4)	–2.853(6)	0.029(4)	0.003(6)
LiH^d^	–7.8787	–7.599(33)	–7.852(62)	0.280(33)	0.029(62)
LiH^e^	–7.8811	–7.360(4)	–7.871(7)	0.521(4)	0.011(7)
BeH_2_^e^	–15.5895	–13.987(5)	–15.563(10)	1.602(5)	0.0263(10)

a Calculations refer to experimental bond distances from the National Institute of Standards and Technology (NIST).^36^ Details on measurement and confidence bounds are provided in the SI.

b Readout mitigation has been applied for all VQE calculations. All energies are given in hartree. Associated standard deviations are provided for all errors.

c Run on Chalmers Särimner with 5000 samples as a single point measurement without optimization. The sample size of 5000 is motivated by our previous experience with the device. The computation on the Särimner device was performed as a complete sweep of the single variational parameter in the circuit, and not as a VQE optimization. Therefore, the energy variance must be approximated through other means, which we describe in the SI.

d Run on ibmq_quito with 8192 samples, the maximum allowed.

e Simulated results using a noise model from ibmq_athens.

Table 1 shows how the application of REM reduces the error by up to 2 orders of magnitude compared to regular VQE when used together with readout mitigation. Without readout mitigation, VQE errors are substantially larger (Tables S3 and S6). The examples in Table 1 are sorted by increasing circuit depth (see also Table 2 for details), which indicate both the robustness and scalability of the approach. The remaining error after mitigation is consistently on the order of millihartree, and the magnitude of the REM correction grows with the complexity of the quantum circuit (cf. H_2_ vs BeH_2_ in Table 1).

**Figure 2:**
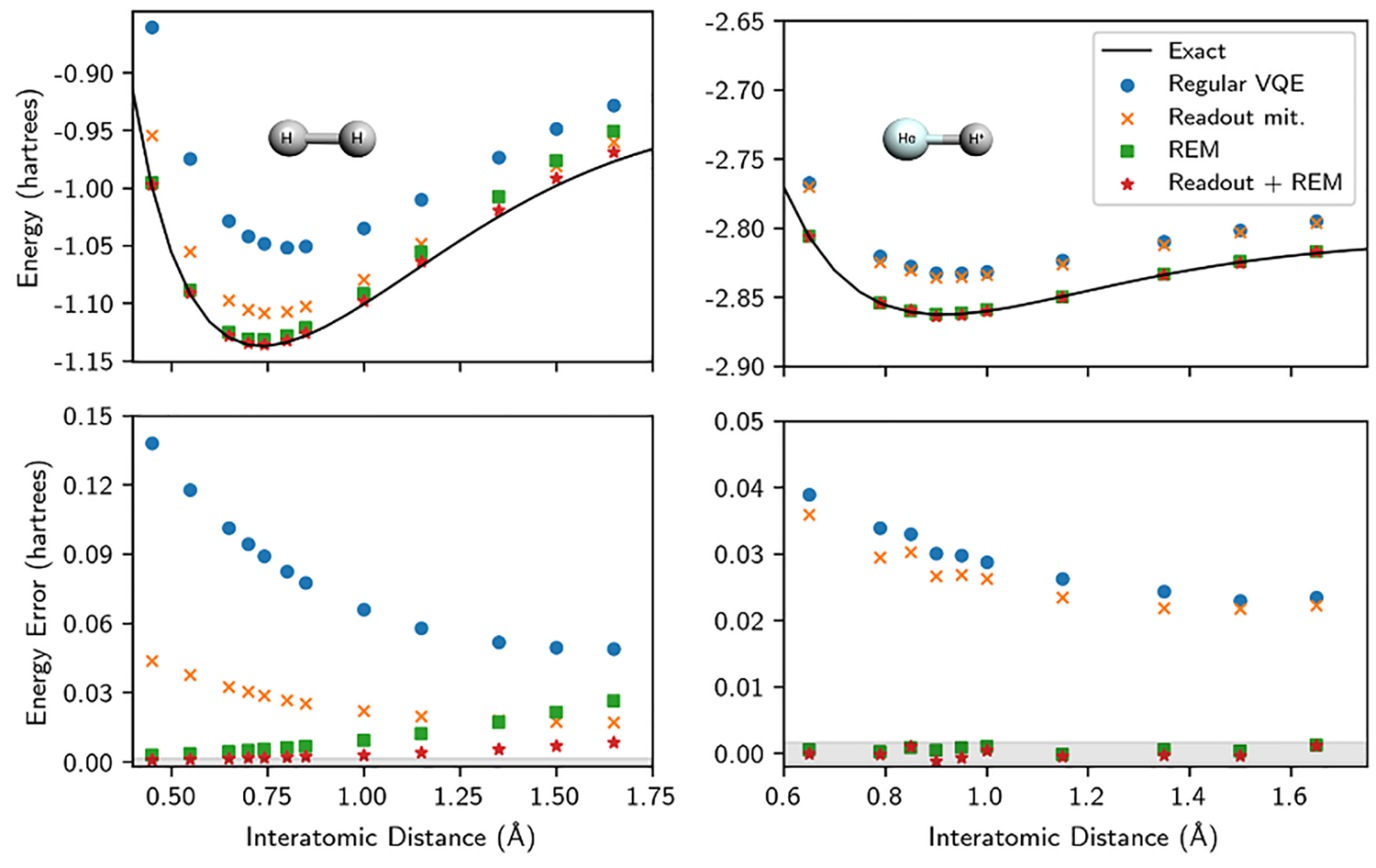
Top: Potential energy surfaces for the dissociation of H_2_ and HeH^+^. Exact noise-free solutions from state-vector simulations are represented by black lines. Regular VQE energies obtained using a quantum computer are shown as blue dots. Results following readout mitigation and REM are shown as orange crosses and green squares, respectively. The combination of both readout mitigation and REM is shown as red stars. Measurements for H_2_ and HeH^+^ were performed on Chalmers Särimner and ibmq_quito, respectively. Bottom: error of the different approaches relative to the exact solution in the given minimal basis set. The gray region corresponds to an error of 1.6 millihartree (1 kcal/mol) with respect to the corresponding noise-free calculations.

**Figure 3:**
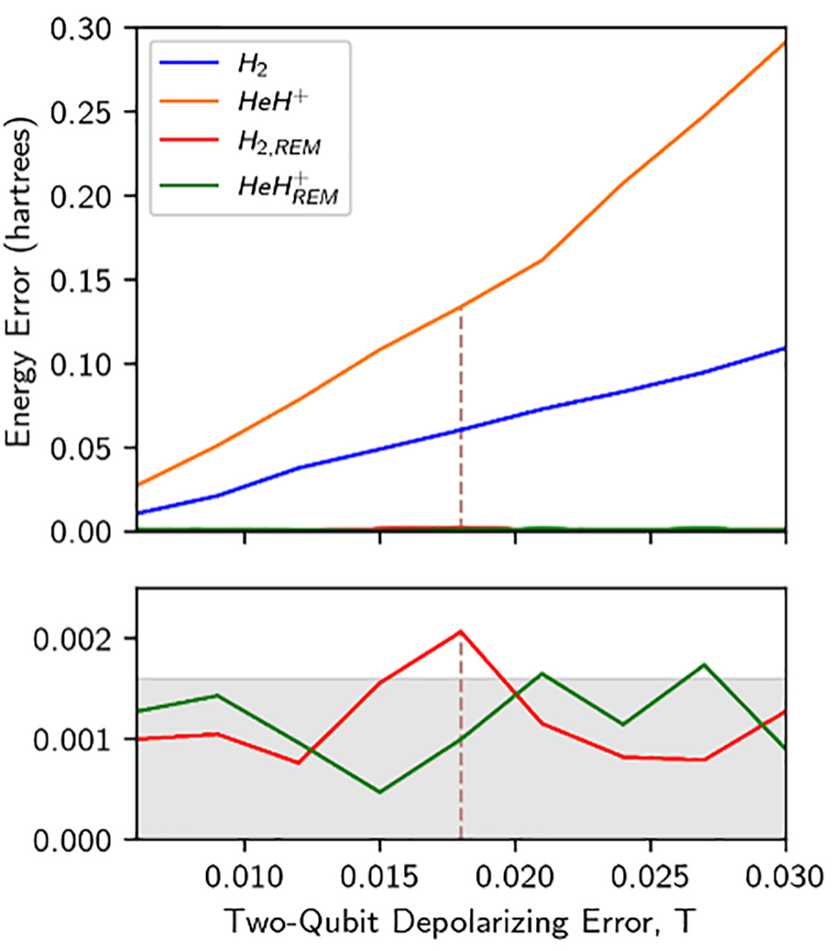
Absolute errors as a function of increasing depolarizing errors for ground state calculations of H_2_ and HeH^+^. The two-qubit depolarizing error of the Chalmers Särimner device is indicated by a vertical line for reference. Bottom inset: Energy errors after application of REM are largely independent of the noise level, and the computational accuracy is consistently close to or below 1.6 millihartree (1 kcal/mol) for these molecules.

In principle, unsuitable choices of reference states combined with significant parameter dependence of noise, Δ*E*_*p*_(*θ⃗*) ≉ 0, might result in overcorrection of the measured VQE energy, taking the energy below the true minimum, as can be seen in some results for the dissociation curve of HeH^+^ (Figures 2 and 3). Nevertheless, we note that REM consistently improves the measured energies, even at relatively high noise levels (Figure 3) and drastically improves the computational accuracy for all calculations summarized in Table 1.

### Testing the Limits of REM

Table 1 demonstrates the effectiveness of REM when applied to molecules in their equilibrium geometry. The test set is small, in practice limited to what is feasible to run on current NISQ hardware. These geometries also represent situations where the degree of electron correlation is relatively low.

To investigate how the REM method performs out of equilibrium, we show in Figure 2 the bond dissociation of H_2_ and HeH^+^. For HeH^+^, which dissociates to He and an isolated proton—a state well described by a single-reference Hartree–Fock description—the REM method provides highly accurate results across the entire binding curve. For H_2_ on the other hand, the effectiveness of the current implementation of REM decreases in regions where the Hartree–Fock state offers a poor description, such as the stretched H_2_ bond. Nevertheless, the method consistently provides a substantial improvement across the potential energy surface. A more suitable description of the partially broken bond of H_2_ should ideally account for the static correlation arising due to near degeneracy of multiple states; i.e., it would require a multireference (MR) or open-shell (OS) reference. We will investigate the use of such MR/OS states and an adaptive choice of the most suitable single reference (SR) state within the REM framework in an upcoming study.

Figure 2 also illustrates the effect of readout mitigation,^17^ which we use per default in all measurements and that we recommend together with REM. Other mitigation strategies may, in principle, also be combined with REM.

The robustness of REM was further evaluated by performing simulations of H_2_ and HeH^+^ while varying the noise level. Noise was introduced in these simulations by modeling imperfect gate-fidelities as single (*S*) and two-qubit (*T*) depolarizing errors, connected through a linear relationship, *S* = 0.1 *T* (see the SI). This kind of noise modeling enables straightforward comparison with error rates on physical quantum devices (Figure 3). REM is shown to be effective despite the steady increase in single- and two-qubit depolarizing error rates.

## Conclusions

In this work we demonstrate an error mitigation strategy applicable to quantum chemical computations on NISQ devices. The REM method relies on accurately determining the error in energy due to hardware and environmental noise for a reference wave function that can be feasibly evaluated on a classical computer. The underlying assumption of REM is a negligible dependence of noise on circuit parameters in the vicinity of this reference wave function. In this work Hartree–Fock references are used, which describes the physics of the molecular states well enough for REM to perform effectively. The REM method is shown to drastically improve the computational accuracy at which total energies of molecules can be computed using current quantum hardware. REM is well suited for calculations with significant amounts of noise and improve calculated energies in all herein tested cases.

The performance of REM is dependent on the quality of the supplied reference state. A Hartree–Fock (mean-field) solution is expected to provide a sufficient reference for molecules that do not exhibit large multireference character. We will investigate the use of references based on multireference and open-shell states, better suited for more strongly correlated problems, in an upcoming study.

In our herein studied problems, the computational accuracy is improved by up to 2 orders of magnitude after application of REM. However, in the presence of substantial quantum circuit parameter dependence of noise it cannot be ruled out that REM may underestimate the true energy. The nonvariational nature of error mitigation strategies remains a problem to be solved. No single mitigation technique will completely resolve the issue of noise, and REM is no exception. One strength of REM is its ability to be combined with other error mitigation techniques without incurring additional cost. REM does not incur meaningful additional classical or quantum computational overhead and can be used to reduce errors on near-term devices by orders of magnitude when running VQE calculations. Because error rates vary both between NISQ devices and between circuits, it is not currently productive to rely on error cancellation, i.e., systematic errors inherent in quantum chemical levels of theory, when evaluating relative energies of chemical transformations. By enabling more precise evaluations of molecular total energies, REM moves us toward meaningful relative comparisons and toward chemical accuracy.

## Appendix: Circuit Complexity Details

Table 2 is presented here.

**Table 2:** Comparison of Circuit Complexities for H_2_, HeH^+^, LiH, and BeH_2_ Listed in Order of Increasing Complexity^a^

Molecule	Ansatz	Qubits	Depth	Two-Qubit Gates	Parameters
H_2_	Simplified UCCD	2	5	1	1
HeH^+^	UCCSD	2	14	4	2
LiH	Hardware-efficient	4	9	6	8
LiH	UCCSD	4	275	172	8
BeH_2_	UCCSD	6	1480	1096	26

a Details are provided in the SI.

## Supporting Information Available

Details of the Chalmers device; description of read out error mitigation; details for H_2_, HeH^+^, LiH, and BeH_2_ calculations, including quantum circuits and Hamiltonians; simulation details for LiH and BeH_2_; calibration details for ibmq_quito device; and description of depolarizing noise model used for simulations (PDF)

## Abbreviations

BeH_2_: beryllium hydride
H_2_: hydrogen molecule
HeH^+^: helium hydride cation
LiH: lithium hydride
NISQ: noisy intermediate-scale quantum
NIST: National Institute for Standards and Technology
REM: reference-state error mitigation
TFLO: training by Fermionic linear optics
VQE: variational quantum eigensolver
MR: multireference
OS: open-shell
SR: single-reference.
