# Supplementary material for: Reference-State Error Mitigation: A Strategy for High Accuracy Quantum Computation of Chemistry
Source: J Chem Theory Comput. 2023 Jan 27;19(3):783–9. doi: 10.1021/acs.jctc.2c00807 (PMC9933421; doi:10.1021/acs.jctc.2c00807)
Supplement: Supplementary file 1 — ct2c00807_si_001.pdf [file ct2c00807_si_001.pdf]

Supporting Information

Reference-State Error Mitigation: A Strategy for High Accuracy Quantum Computation of Chemistry

Phalgun Lolur[1], Mårten Skogh[1,2], Werner Dobrautz[1], Christopher Warren[3], Janka Biznárová[3], Amr Osman[3], Giovanna Tancredi[3], Göran Wendin[3], Jonas Bylander[3] and Martin Rahm[1,*]

1. Department of Chemistry and Chemical Engineering, Chalmers University of Technology, SE-412 96 Gothenburg, Sweden
2. Data Science & Modelling, Pharmaceutical Science, R&D, AstraZeneca, Gothenburg, SE-431 83 Mölndal, Sweden
3. Department of Microtechnology and Nanoscience MC2, Chalmers University of Technology, SE-412 96 Gothenburg, Sweden

* Corresponding author: martin.rahm@chalmers.se

Table of Content

Notation 2

Chalmers Device Details 3

Readout Error Mitigation 5

Hydrogen – H_2 6

Ansatz, circuit and computation details 6

H_2 Hamiltonian 8

Data 9

Helium hydride – HeH^+ 11

Ansatz, circuit and computation details 11

HeH^+ Hamiltonian 12

Data 13

Lithium hydride – LiH 15

Ansatz, circuit and computation details 15

LiH Hamiltonian details at 1.5949 Å 16

Data 17

Simulation Details for Lithium Hydride (LiH) and Beryllium Hydride (BeH_2) 18

IBM-Quito Device calibration and connectivity details 19

Depolarizing Noise Model 20

References 21

Notation

The following notation is used in the SI:

$E_{exact}(\vec{\theta}_{ref})$ – Energy of the reference state in the absence of noise

$E_{VQE}(\vec{\theta}_{ref})$ – Energy of the reference state from VQE

$E_{VQE*}(\vec{\theta}_{ref})$ – Energy of the reference state from VQE with readout mitigation

$E_{exact}(\vec{\theta}_{min})$ – Energy of the state of interest in the absence of noise

$E_{VQE}(\vec{\theta}_{min,VQE})$ – Energy of the state of interest from VQE

$E_{VQE*}(\vec{\theta}_{min,VQE})$ – Energy of the state of interest from VQE with readout mitigation

E_{REM} – Energy of the state of interest from VQE with REM

E_{REM*} – Energy of the state of interest from VQE with readout mitigation and REM

$\Delta E_{error,VQE}$ – Error of the VQE energy with respect to $E_{exact}(\vec{\theta}_{\mathrm{ref}})$

$\Delta E_{error,VQE*}$ – Error of the readout mitigated VQE energy with respect to $E_{\mathrm{exact}}(\vec{\theta}_{\mathrm{ref}})$

$\Delta E_{error,REM}$ – Error of the REM mitigated VQE energy with respect to $E_{\mathrm{exact}}(\vec{\theta}_{\mathrm{ref}})$

$\Delta E_{error,REM*}$ – Error of the REM+readout mitigated VQE energy with respect to $E_{exact}(\vec{\theta}_{ref})$

r – Interatomic distance (Å)

Chalmers Device Details

We executed the quantum algorithm at Chalmers on a superconducting three-qubit quantum processor named Särimner, of which we only use two qubits (Q_0, Q_1). This device is shown in Figure S1 and consists of three transmon qubits,[1] coupled using a single tunable coupler, C_1. Single-qubit gates are implemented using on-chip drive lines to individually control each qubit with microwave pulses. Each qubit can be individually measured using its readout resonator with readout performed simultaneously using frequency multiplexed pulses on the common readout feedline. Two-qubit gates are activated via an AC flux-pulse applied to the coupler to modulate its frequency.[2] The coupler is itself a frequency-tunable transmon qubit, however, it only serves to mediate the interaction between pairs of qubits and itself never enters the computational space during operation. A full list of parameters of the device can be found in Table S1.

The AC flux-pulse which activates the coupling takes the form $\Phi(t) = \Phi_b + \Omega(t)\cos(\omega_d t)$, where Φ_b is the DC flux bias of the drive, $\Omega(t)$ is the envelope of the pulse which consists of a 25 ns cosine rise and fall and a 310 ns flat top, and ω_d is the carrier frequency. The carrier frequency is chosen such that it drives a controlled-Z (CZ) transition between Q_0 and Q_1. This is on resonance with the transition $|20\rangle \leftrightarrow |11\rangle$ and occurs at $\omega_d = |\omega_0 + \eta_0 - \omega_1|$. A full oscillation between these states brings about a conditional phase on the $|11\rangle$ state which can be calibrated to implement a CZ gate.

The parametric gate is useful in this higher connectivity architecture as the interaction between pairs can be selectively chosen. Different gates are activated between qubits when the frequencies between transitions for pairs of qubits are off-resonant from one another as they are in this device. The third qubit can also be detuned such that all transitions are far off-resonant and there is no risk of a frequency collision when operating the device with just Q_0 and Q_1.

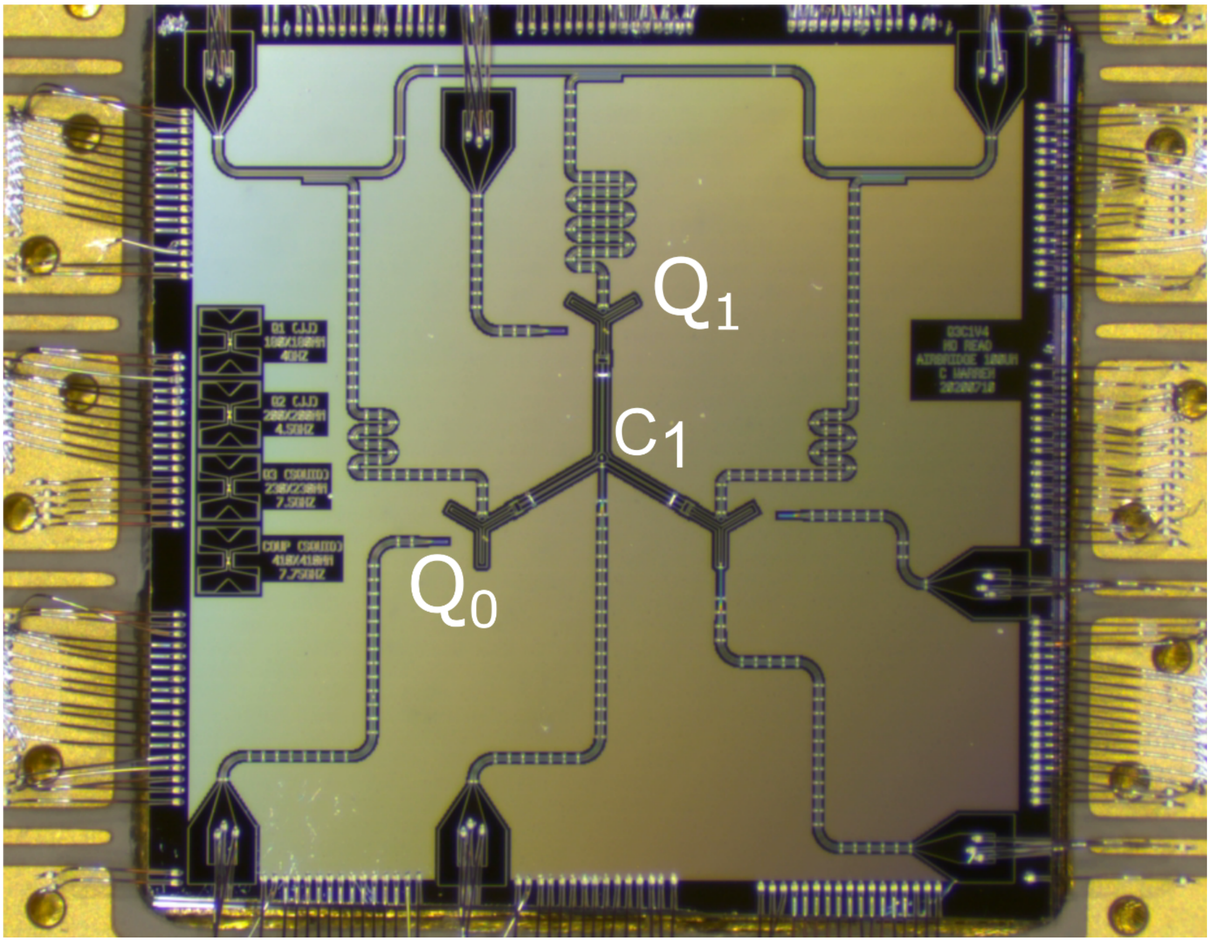

Figure S1: Microscope image of the bonded 3-qubit Särimner device from Chalmers. Only Q_0 and Q_1 are used in this work. The coupler, C_1, consists of a flux-tunable transmon qubit and serves to mediate interactions between pairs. Each qubit has individual readout and drive lines which are used to control the device. The readout lines are coupled to a shared feedline which is used for multiplexed readout.

Table S1: Experimental parameters for the 3-qubit Chalmers device Särimner. We report our average gate error achieved through randomized benchmarking for single qubit gates, as our gate set differs from that of IBM, as well as interleaved randomized benchmarking for our 2-qubit CZ gate.

Qubit	T_1 [μs]	T_2 [μs]	Qubit Frequency, ω_i [GHz]	Anharmonicity, η_i [GHz]	Single Qubit Gate Error	CZ-Gate Error
Q_0	35.98	38.74	3.799	-0.1885	4.9e-4	1.8e-2
Q_1	36.24	39.34	4.383	-0.1837	5.2e-4	1.8e-2

The single- and two-qubit gate fidelities during the execution of the quantum algorithm were 99.95% and 98.2%. The device design, fabrication methods, measurement setup, gate implementation on hardware, and tune-up methods are described in detail elsewhere.[3]

Readout Error Mitigation

Readout error mitigation is performed by constructing a calibration of the confusion matrix C. The entries of this matrix, $C_{i,j}$, are the probabilities of preparing the state $|i\rangle$ and then measuring the state $|j\rangle$, i.e., $C_{i,j} = P(\, j \mid i\,)$. The matrix C can then be used to correct a set of Pauli string measurements $\vec{m} = [P(0), \dots, P(j), \dots, P(n)]^{\mathrm{T}}$ by either multiplying by the inverse confusion matrix, $C^{-1}\vec{m} = \vec{m}'$, performing a least-squares fit to reconstruct the most likely outcome, or by a procedure known as 'Bayesian Unfolding'.[4]

In this work, we mitigate our results by implementing a least-square fit to a quadratic cost function, where $\lambda(\vec{x}) = (\vec{m} - C\vec{x})^2$ with the constraint that the sum of the resulting vector must be 1 and each element itself is bounded in the interval [0,1]. This avoids some issues resulting from matrix inversion arising from small off-diagonal elements, which can lead to unphysical results. The Sequential Least Squares Programming[5] (SLSQP) optimizer was used to find the $\vec{x}$ that minimize the cost for each set of measured Pauli strings. The confusion matrix of the Chalmers Särimner device is reported in Figure S2.

Both options for readout mitigation are also available in Qiskit and can be straightforwardly implemented for calculations on real devices and simulations.

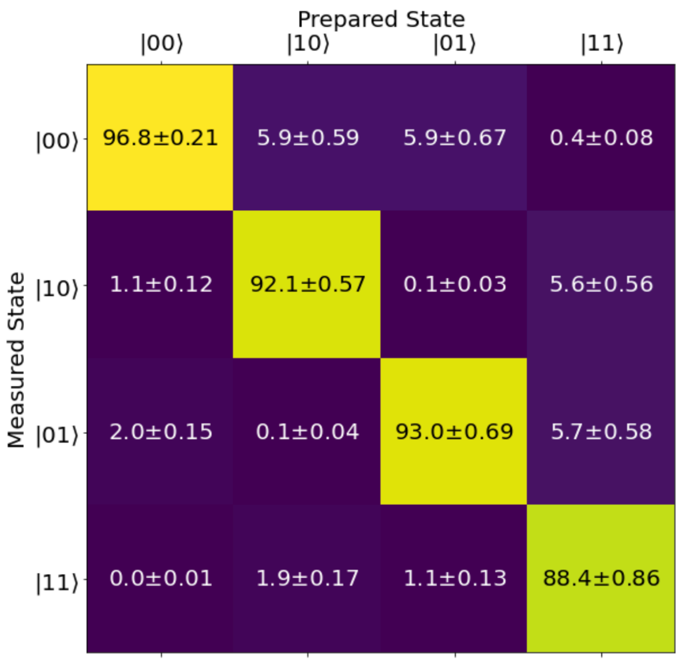

Figure S2. Confusion matrix for the Chalmers Särimner device. The matrix consists of probabilities of measuring a state given a specific preparation. The confusion matrix is measured before each run of the VQE algorithm with 1000 shots and repeated 100 times to give an estimate for fluctuations in the readout.

General Computational Details

For all calculations described below, PySCF[6] was used to generate the initial Hartree-Fock state, utilizing the STO-3G basis set. The circuit ansatz was constructed using a parameterized wavefunction based on unitary coupled cluster theory[7] as implemented in Qiskit 0.21.[8] Additionally, parity mapping[7] was used to map the fermionic spin-orbital occupation to qubits. Two-particle reduction was used to reduce the problem by two qubits in all cases, corresponding to alpha and beta spin parity conservation. For the optimization in the VQE algorithm the COBYLA optimizer was used. Unless specifically stated, default Qiskit 0.21 settings and parameters were used.

Hydrogen – H_2

Ansatz, circuit and computation details

The H_2 wavefunction can be represented with four qubits, where each qubit corresponds to one molecular spin orbital in minimal STO-3G basis. Since single excitations do not contribute to the final ground state energy of H_2, only double excitations were included in the ansatz which reduced the complexity further, to a single parameter, θ. The resulting circuit, compiled to Qiskit's native U1, U2 and U3 gates, is depicted in Figure S3.

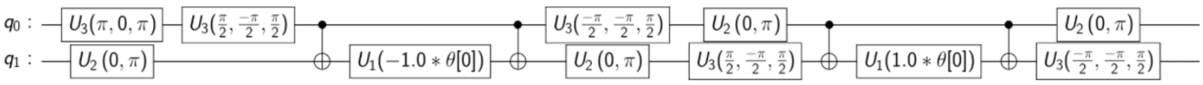

Figure S3: Quantum circuit for H_2 implementing double excitation of the unitary coupled-cluster operator with respect to the Hartree-Fock reference state. The circuit is compiled to the native gates of the IBMQ-Quito device.

The circuit shown in in Figure S3 was then shortened further by removing a repeated entangling step. A single entangling step was found sufficient to explore the Hilbert space of the problem. The resulting circuit was transpiled to gates native to the Särimner device (Figure S4) and is similar to the one used by Kandala et. Al.[9] The Särimner gate set consists of the set of single-qubit gates, $\left\{R_x(\pm\pi), R_y(\pm\pi), R_x\left(\pm\frac{\pi}{2}\right), R_y\left(\pm\frac{\pi}{2}\right), R_z(\theta)\right\}$, and two-qubit gate set $\{\text{CZ}\}$. Both circuits return the same energy up to the eighth decimal point of a hartree numerically, justifying our circuit design. Since the circuit contains only one parameter, it was varied in the interval [-π, π] to obtain energy as a function of the variational parameter at several geometries. The obtained energies were fit using the lmfit package[10] into a cosine function, A $\cos(\theta - \alpha)$, where A and α are fit parameters, to give the minima at different geometries (Figure S5). All experimental calculations were run on the Chalmers Särimner device with 5000 shots. The specific number of shots were chosen based on previous experience with the device. The details of the Hamiltonian operator to be minimized are given in Table S2 for different geometries of H_2. The exact solutions for the given circuit and Hamiltonians were calculated using QuTiP.[11]

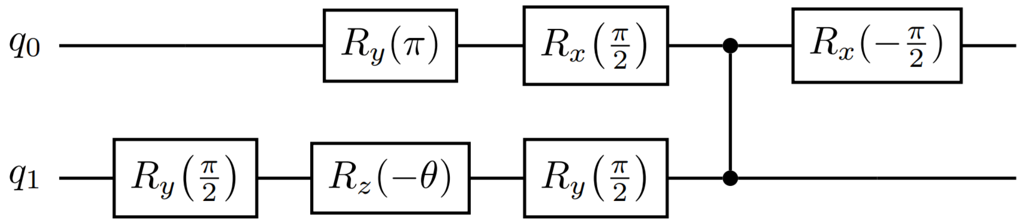

Figure S4: A compact quantum circuit for H_2 implementing double excitation of the coupled-cluster operator with respect to the Hartree-Fock reference state. The circuit is compiled to the native gates of Chalmers Särimner device.

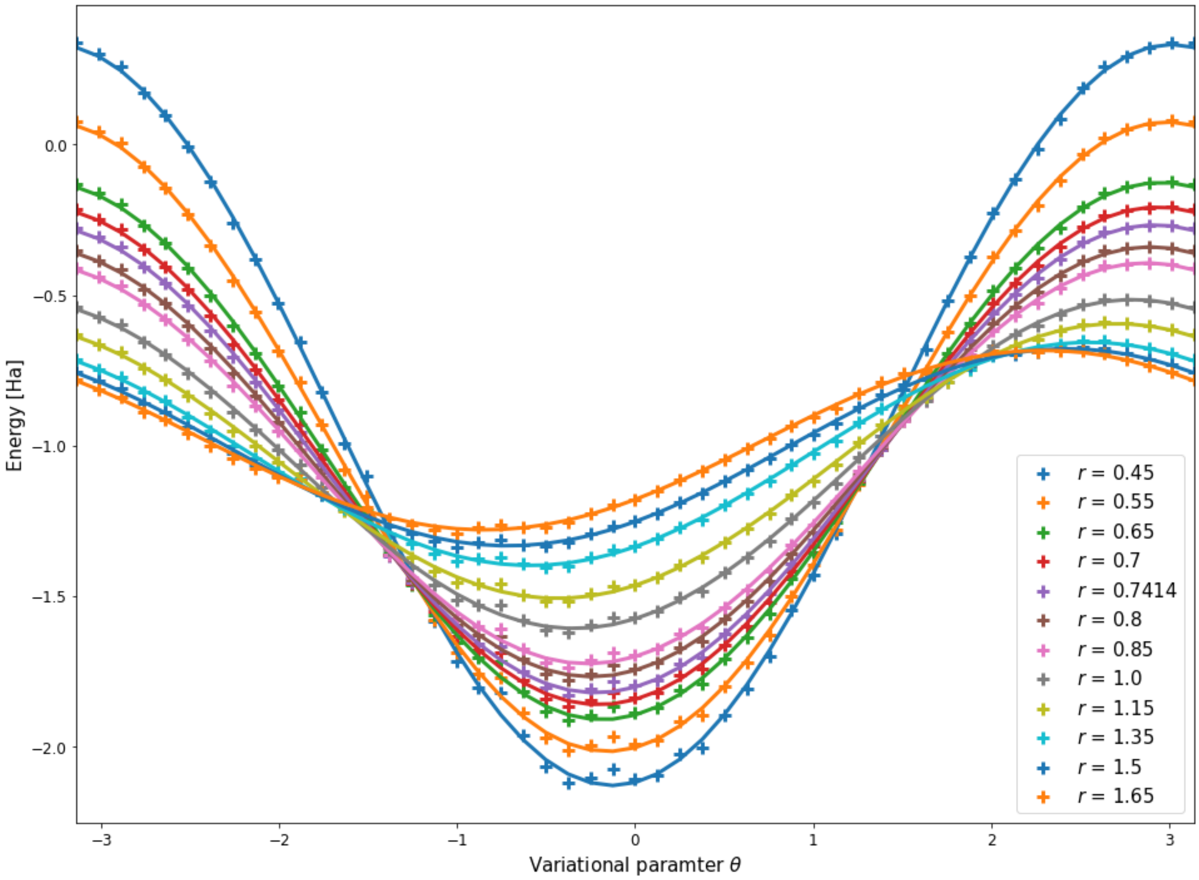

Figure S5: Measurement results for a single sweep of θ between $-\pi$ and π. The same measurement results have been used with different electronic Hamiltonians to generate the energies using QuTiP[11] for the different geometries. A cosine function of the form, A $\cos(\theta - \alpha)$, is used to generate the fits for the data.

H_2 Hamiltonian

Table S2: The electronic Hamiltonian for H_2 expressed as weighted Pauli string operators after parity mapping for various geometry.

r	II	IZ	ZI	ZZ	XX
0.45	-0.908	0.634	-0.634	-0.013	0.167
0.55	-0.981	0.536	-0.536	-0.012	0.171
0.65	-1.028	0.455	-0.455	-0.012	0.176
0.70	-1.044	0.420	-0.420	-0.012	0.179
0.7414	-1.054	0.394	-0.394	-0.011	0.181
0.80	-1.063	0.360	-0.360	-0.011	0.185
0.85	-1.068	0.334	-0.334	-0.010	0.188
1.00	-1.069	0.268	-0.268	-0.009	0.197
1.15	-1.058	0.215	-0.215	-0.007	0.206
1.35	-1.033	0.161	-0.161	-0.005	0.220
1.50	-1.010	0.129	-0.129	-0.004	0.230
1.65	-0.985	0.103	-0.103	-0.003	0.239

Data

The total energy of a system is the sum of its electronic and nuclear repulsion energies at a given geometry for H_2. The optimal parameters are reported in Table S3. The nuclear energies are reported in Table S4, and the electronic energies are reported in Table S5.

Table S3: Optimized parameters, before and after readout mitigation, that minimize the Hamiltonian operator on the Chalmer Särimner device for calculations of H_2.

r	θ (Uncorrected)	θ (Readout mit.)
0.45	-0.1186	-0.1272
0.55	-0.1437	-0.1540
0.65	-0.1737	-0.1861
0.70	-0.1906	-0.2042
0.7414	-0.2056	-0.2202
0.80	-0.2284	-0.2445
0.85	-0.2495	-0.2669
1.00	-0.3220	-0.3438
1.15	-0.4106	-0.4372
1.35	-0.5553	-0.5876
1.50	-0.6802	-0.7153
1.65	-0.8121	-0.8477

Table S4: Nuclear repulsion energies (V_{NN}) of H_2 at various geometries, r. All V_{NN} energies in hartrees.

r	V_{NN}
0.45	1.1759
0.55	0.9621
0.65	0.8141
0.70	0.7560
0.7414	0.7138
0.80	0.6615
0.85	0.6226
1.00	0.5292
1.15	0.4602
1.35	0.3920
1.50	0.3528
1.65	0.3207

Table S5: Exact, regular VQE and mitigated electronic energies and errors of H_2 at various geometries, r. All energies are in hartrees.

r [Å]	$E_{exact}(\vec{\theta}_{ref}$	$E_{VQE}(\vec{\theta}_{ref})$	$E_{VQE*}(\vec{\theta}_{ref})$	$E_{exact}(\vec{\theta}_{min}$	$E_{VQE}(\vec{\theta}_{min,VQE})$	$E_{VQE*}(\vec{\theta}_{min,VQE}$	E_{REM}	E_{REM*}	$\Delta E_{error,VQE}$	$\Delta E_{error,VQE*}$	$\Delta E_{error,REM}$	$\Delta E_{error,REM*}$
0.45	-0.9875	-0.8524	-0.9446	-0.9984	-0.8604	-0.9546	-0.9955	-0.9975	0.1380	0.0438	0.0029	0.0010
0.55	-1.0791	-0.9649	-1.0426	-1.0926	-0.9749	-1.0550	-1.0890	-1.0914	0.1178	0.0376	0.0036	0.0012
0.65	-1.1130	-1.0162	-1.0820	-1.1299	-1.0287	-1.0974	-1.1254	-1.1284	0.1013	0.0325	0.0045	0.0015
0.70	-1.1173	-1.0281	-1.0886	-1.1362	-1.0419	-1.1058	-1.1312	-1.1345	0.0943	0.0304	0.0050	0.0017
0.7414	-1.1167	-1.0331	-1.0897	-1.1373	-1.0482	-1.1085	-1.1318	-1.1355	0.0891	0.0288	0.0055	0.0018
0.80	-1.1109	-1.0345	-1.0861	-1.1341	-1.0516	-1.1073	-1.1280	-1.1321	0.0825	0.0268	0.0062	0.0020
0.85	-1.1025	-1.0317	-1.0794	-1.1284	-1.0507	-1.1030	-1.1215	-1.1261	0.0776	0.0253	0.0068	0.0023
1.00	-1.0661	-1.0093	-1.0473	-1.1012	-1.0352	-1.0793	-1.0919	-1.0981	0.0660	0.0219	0.0092	0.0030
1.15	-1.0210	-0.9752	-1.0054	-1.0679	-1.0099	-1.0483	-1.0557	-1.0639	0.0580	0.0196	0.0122	0.0040
1.35	-0.9572	-0.9227	-0.9449	-1.0251	-0.9733	-1.0072	-1.0078	-1.0195	0.0517	0.0179	0.0172	0.0056
1.50	-0.9109	-0.8830	-0.9005	-0.9981	-0.9486	-0.9808	-0.9765	-0.9912	0.0495	0.0173	0.0216	0.0070
1.65	-0.8678	-0.8452	-0.8590	-0.9771	-0.9283	-0.9599	-0.9508	-0.9688	0.0489	0.0172	0.0263	0.0084

Helium hydride – HeH^+

Ansatz, circuit and computation details

Similar to H_2, the HeH^+ wavefunction can be represented with two qubits using parity mapping[7] in a minimal basis. The circuit consists of three parameters – two single excitation parameters, *θ[0]* and *θ[1]*, and a double excitation parameter, *θ[2].* These parameters are optimized using the VQE algorithm implemented in Qiskit and run on the IBMQ-Quito device with 8192 shots, the maximum number of allowed shots we had access to on IBMQ. The details of the Hamiltonian operator to be minimized are given in Table S6 for different geometries of HeH^+.

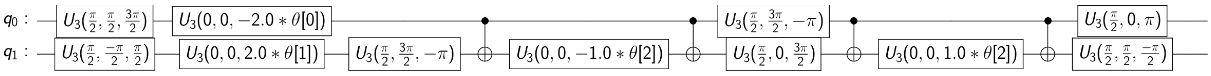

Figure S6: Quantum circuit for HeH^+ implementing the UCCSD operator with respect to the Hartree-Fock reference state. The circuit is compiled to the native gates of IBMQ-Quito device.

HeH^+ Hamiltonian

The details of the Hamiltonian operator to be minimized are given in Table S6 for different geometries of HeH^+.

Table S6: The electronic Hamiltonian expressed as weighted Pauli string operators after parity mapping for various geometry of HeH^+.

r	II	IZ	ZI	ZZ	ZX	XZ	IX	XI	XX
0.65	-3.229	0.635	-0.635	-0.074	-0.094	0.094	0.094	0.094	0.157
0.7899	-3.161	0.560	-0.560	-0.097	-0.106	0.106	0.106	0.106	0.144
0.85	-3.129	0.538	-0.538	-0.108	-0.111	0.111	0.111	0.111	0.137
0.90	-3.101	0.523	-0.523	-0.118	-0.114	0.114	0.114	0.114	0.131
0.95	-3.073	0.512	-0.512	-0.128	-0.117	0.117	0.117	0.117	0.124
1.00	-3.045	0.503	-0.503	-0.139	-0.119	0.119	0.119	0.119	0.117
1.15	-2.962	0.488	-0.488	-0.173	-0.122	0.122	0.122	0.122	0.095
1.35	-2.857	0.488	-0.488	-0.217	-0.115	0.115	0.115	0.115	0.066
1.5	-2.785	0.495	-0.495	-0.247	-0.104	0.104	0.104	0.104	0.047
1.65	-2.721	0.506	-0.506	-0.273	-0.090	0.090	0.090	0.090	0.032

Data

The total energy of a system is the sum of its electronic and nuclear repulsion energies at a given geometry for H_2. The optimal parameters are reported in Table S7. The nuclear energies are reported in Table S8, and the electronic energies are reported in Table S9.

Table S7: The optimized parameter values that minimize the Hamiltonian operator on the IBMQ-Quito device for various geometry of HeH^+. The angles are in radians.

r	$\theta[0]$	$\theta[1]$	$\theta[2]$
0.65	0.011	0.008	-0.061
0.7899	0.014	0.016	-0.067
0.85	0.013	0.010	-0.065
0.90	0.012	0.013	-0.063
0.95	0.017	0.015	-0.065
1.00	0.021	0.021	-0.063
1.15	0.017	0.017	-0.053
1.35	0.012	0.012	-0.036
1.5	0.009	0.003	-0.025
1.65	0.008	0.005	-0.018

Table S8: Nuclear repulsion energies (V_{NN}) of HeH^+ at various geometries, r. All V_{NN} energies in hartrees.

r	V_{NN}
0.65	1.6282
0.7899	1.3399
0.85	1.2451
0.90	1.1759
0.95	1.1141
1.00	1.0584
1.15	0.9203
1.35	0.7840
1.5	0.7056
1.65	0.6414

Table S9: The exact, uncorrected and mitigated electronic energies and errors of HeH^+ at various geometries, r. All energies are in hartrees.

r [Å]	$E_{exact}(\vec{\theta}_{ref})$	$E_{VQE}(\vec{\theta}_{ref})$	$E_{VQE*}(\vec{\theta}_{ref})$	$E_{exact}(\vec{\theta}_{min})$	$E_{VQE}(\vec{\theta}_{min,VQE})$	$E_{VQE*}(\vec{\theta}_{min,VQE})$	E_{REM}	E_{REM*}	$\Delta E_{error,VQE}$	$\Delta E_{error,VQE*}$	$\Delta E_{error,REM}$	$\Delta E_{error,REM*}$
0.65	-2.7964	-2.7580	-2.7604	-2.8062	-2.7673	-2.7703	-2.8057	-2.8063	0.0389	0.0359	0.0005	-0.0001
0.7899	-2.8447	-2.8110	-2.8150	-2.8542	-2.8203	-2.8247	-2.8540	-2.8544	0.0338	0.0294	0.0002	-0.0002
0.85	-2.8517	-2.8195	-2.8225	-2.8608	-2.8278	-2.8305	-2.8600	-2.8597	0.0330	0.0302	0.0008	0.0010
0.90	-2.8540	-2.8244	-2.8261	-2.8626	-2.8326	-2.8359	-2.8622	-2.8638	0.0300	0.0267	0.0004	-0.0012
0.95	-2.8542	-2.8253	-2.8267	-2.8622	-2.8324	-2.8353	-2.8614	-2.8629	0.0298	0.0269	0.0008	-0.0007
1.00	-2.8529	-2.8252	-2.8270	-2.8602	-2.8315	-2.8339	-2.8592	-2.8598	0.0287	0.0263	0.0010	0.0004
1.15	-2.8445	-2.8181	-2.8206	-2.8495	-2.8233	-2.8261	-2.8497	-2.8500	0.0262	0.0235	-0.0002	-0.0004
1.35	-2.8314	-2.8076	-2.8093	-2.8339	-2.8095	-2.8120	-2.8333	-2.8341	0.0243	0.0219	0.0005	-0.0003
1.5	-2.8234	-2.8008	-2.8013	-2.8247	-2.8017	-2.8029	-2.8244	-2.8251	0.0230	0.0218	0.0003	-0.0004

Lithium hydride – LiH

Ansatz, circuit and computation details

A LiH wavefunction can be represented with twelve qubits where each qubit corresponds to one molecular spin orbital in a minimal STO-3G basis. Symmetries in parity mapping[7] was used to further reduce the problem size by two qubits to ten qubits. As shown by Kandala *et al*[9], removing the orbitals that do not participate in bonding can bring down the problem size by four qubits. Coupled with the frozen core approximation, the final LiH circuit can be represented by just four qubits in and around equilibrium geometry. A hardware efficient ansatz, inspired by Qiskit's two-local circuit class is used to construct a compact circuit representing LiH. It consists of alternating rotating layers of entanglement layers and is chosen to utilize the connectivity of IBMQ-Quito's connectivity as shown in Figure S7.

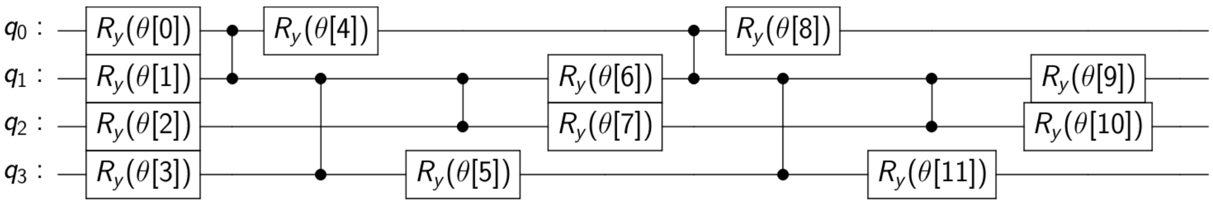

Figure S7: Quantum circuit for LiH utilizing a hardware-efficient ansatz.

LiH Hamiltonian details at 1.5949 Å

Table S10: The electronic Hamiltonian of LiH can be expressed as weighted Pauli string operators with the following coefficients:

IIII	-0.207	ZXIZ	0.012	XZIZ	-0.013	YYXZ	0.008
IIIZ	-0.094	IXZX	-0.003	XIZX	-0.002	XXXZ	-0.008
IIZX	-0.003	ZXZX	-0.003	XZZX	0.002	YYXI	0.008
IIIX	0.003	IXIX	0.003	XIIX	0.002	XXXI	-0.008
IIXX	-0.001	ZXIX	0.003	XZIX	-0.002	ZZZZ	0.084
IIYY	0.001	IXXX	-0.009	XIXX	-0.008	ZZXZ	-0.009
IIZZ	-0.212	ZXXX	-0.009	XZXX	0.008	ZZXI	-0.009
IIXZ	0.019	IXYY	0.009	XIYY	0.008	XIZZ	-0.009
IIXI	0.019	ZXYY	0.009	XZYY	-0.008	XZZZ	0.009
IIZI	0.359	YYIZ	0.032	ZIIZ	0.114	XIXZ	0.007
IZII	0.094	XXIZ	-0.032	ZIZX	-0.011	XZXZ	-0.007
ZXII	0.003	YYZX	-0.009	ZIIX	0.011	XIXI	0.007
IXII	0.003	XXZX	0.009	ZIXX	-0.034	XZXI	-0.007
XXII	-0.001	YYIX	0.009	ZIYY	0.034	ZIZZ	0.060
YYII	0.001	XXIX	-0.009	IZZZ	-0.056	ZIXZ	0.011
ZZII	-0.212	YYXX	-0.031	IZXZ	-0.013	ZIXI	0.011
XZII	-0.019	XXXX	0.031	IZXI	-0.013	IZZI	0.114
XIII	0.019	YYYY	0.031	IXZZ	-0.002	IXZI	-0.011
ZIII	-0.359	XXYY	-0.031	ZXZZ	-0.002	ZXZI	-0.011
IZIZ	-0.122	ZZIZ	0.056	IXXZ	0.002	YYZI	-0.034
IZZX	0.012	ZZZX	0.002	ZXXZ	0.002	XXZI	0.034
IZIX	-0.012	ZZIX	-0.002	IXXI	0.002	ZZZI	-0.060
IZXX	0.032	ZZXX	0.003	ZXXI	0.002	XIZI	-0.011
IZYY	-0.032	ZZYY	-0.003	YYZZ	-0.003	XZZI	0.011
IXIZ	0.012	XIIZ	0.013	XXZZ	0.003	ZIZI	-0.113

Data

Frozen core energy: -7.7983328 hartrees

Nuclear repulsion energy: 0.99538004 hartrees

Table S11: The exact, uncorrected, and mitigated electronic energies and errors of LiH at 1.5949 Å. All energies are in hartrees.

$E_{exact}(\vec{\theta}_{ref})$	$E_{VQE}(\vec{\theta}_{ref})$	$E_{VQE*}(\vec{\theta}_{ref})$	$E_{exact}(\vec{\theta}_{min})$	$E_{VQE}(\vec{\theta}_{min,VQE})$	$E_{VQE*}(\vec{\theta}_{min,VQE})$	E_{REM}	E_{REM*}	$\Delta E_{error,VQE}$	$\Delta E_{error,VQE*}$	$\Delta E_{error,REM}$	$\Delta E_{error,REM}$
-7.8620	-7.6064	-7.6071	-7.8787	-7.6071	-7.6102	-7.8627	-7.8651	0.2717	0.2686	0.0160	0.0136

Optimal Parameters:

Table S12: The optimized parameter values that minimize the Hamiltonian operator on the IBMQ-Quito device for LiH at 1.5949 Å. The angles are in radians.

$\theta[0]$	$\theta[1]$	$\theta[2]$	$\theta[3]$	$\theta[4]$	$\theta[5]$	$\theta[6]$	$\theta[7]$	$\theta[8]$	$\theta[9]$	$\theta[10]$	$\theta[11]$
3.8987	-6.5469	-1.2442	-5.0653	1.5509	2.0379	3.1205	-4.7523	2.3617	6.2591	-5.9394	3.2559

Simulation Details for Lithium Hydride (LiH) and Beryllium Hydride (BeH_2)

As shown in the previous section, LiH wavefunction can be represented with four qubits. Making similar approximations as shown by Kandala *et al*[9], the BeH_2 wavefunction can be represented by six qubits when using the frozen core approximation and orbital reduction. Qiskit's UCCSD module is used to construct the problem ansatz. The circuits are too large to be represented here but the details of the circuit can be found in Table A1. A noise-model from IBMQ-Athens has been added to the simulations to replicate real-world noisy behavior. All simulations have been run using 20,000 shots and repeated 5 times to ensure a high number of samples. The sampling noise is expressed in terms of the standard deviations of our errors. Readout mitigation has been applied for all the simulations.

Table S13: Total ground state energies of molecules(simulated) at experimental equilibrium distances, without and with the application of REM. Bond distances have been obtained from the National Institute of Standards and Technology (NIST). A noise model from IBMQ-Athens has been added to all the simulations. Readout mitigation has been applied for all the VQE calculations. All energies are given in Hartree. The sampling error of the simulations is represented as the standard deviation.

Molecule	$E_{exact}(\vec{\theta}_{min})$	$E_{VQE} * (\vec{\theta}_{min,VQE})$	E_{REM*}	$\Delta E_{error,VQE} *$	$\Delta E_{error,REM*}$
LiH	-7.8811	-7.3599	-7.8705	0.5213 ± 0.003	0.0106 ± 0.002
BeH_2	-15.5895	-13.9873	-15.5632	1.6021 ± 0.005	0.0263 ± 0.007

IBM-Quito Device calibration and connectivity details

Table S14: IBM-Quito's calibration details, as imported from IBM Quantum Services. The device reports a quantum volume of 32.

Qubit	T1 (μs)	T2 (μs)	Frequency (GHz)	Anharmonicity (GHz)	Readout assignment error	Prob meas0 prep1	Prob meas1 prep0	Readout length (ns)	ID error	Single-qubit Pauli-X error	CNOT error	Gate time (ns)
Q0	85.96	95.63	5.300	-0.33148	0.0300	0.0468	0.0132	5351.111	$2.93 \cdot 10^{-4}$	$2.93 \cdot 10^{-4}$	0_1:$6.276 \cdot 10^{-3}$	0_1:234.667
Q1	125.84	128.97	5.081	-0.31925	0.0134	0.0208	0.0060	5351.111	$2.92 \cdot 10^{-4}$	$2.92 \cdot 10^{-4}$	1_3:$1.079 \cdot 10^{-2}$;<br>1_2:$7.076 \cdot 10^{-3}$;<br>1_0:$6.276 \cdot 10^{-3}$	1_3:334.222;<br>1_2:298.667;<br>1_0:270.222
Q2	81.75	123.32	5.322	-0.33232	0.0237	0.0358	0.0116	5351.111	$2.55 \cdot 10^{-4}$	$2.55 \cdot 10^{-4}$	2_1:$7.076 \cdot 10^{-3}$	2_1:263.111
Q3	96.32	10.29	5.164	-0.33508	0.0281	0.0454	0.0108	5351.111	$3.13 \cdot 10^{-4}$	$3.13 \cdot 10^{-4}$	3_4:$1.557 \cdot 10^{-3}$;<br>3_1:$1.079 \cdot 10^{-2}$	3_4:277.333;<br>3_1:369.778
Q4	148.13	162.06	5.052	-0.31926	0.0208	0.0328	0.0088	5351.111	$2.74 \cdot 10^{-4}$	$2.74 \cdot 10^{-4}$	4_3:$1.557 \cdot 10^{-2}$	4_3:312.889

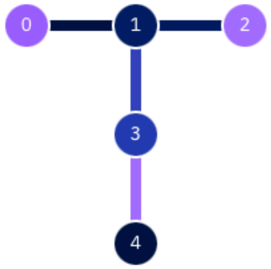

Figure S8: Connectivity representation of the IBMQ-Quito[12] device.

Depolarizing Noise Model

Depolarizing noise channels are a common way to model decoherence and gate errors in quantum devices.[13,14] After observing that our two-qubit (T) gate-error is approximately an order of magnitude larger than our single-qubit (S) gate error, a noise model was constructed that provides S as a linear function of T, $S = 0.1\,T$. This noise model was used to evaluate the effect of REM, applied to a range of depolarizing errors. The depolarizing noise was modelled in Qiskit as a probability p' of applying each of the Pauli gates X, Y, or Z after running a single-qubit gate, and as probability p'' of applying any combination of $P_i \otimes P_j$ where $P_i, P_j \in \{I, X, Y, Z\}$ after running a two-qubit gate.

Uncertainty due to Sampling

The uncertainty associated with REM is due to the limited number of samples that can be measured for each point on the noisy energy surface. The resulting spread in the result is characterized by its variance, $\mathrm{Var}(E(\vec{\theta}))$. Assuming that the measurements are normally distributed, the variance of the REM results are inherently larger than the unmitigated ones, as we need to sample two independent distributions, both for the VQE and the reference calculation. Thus, we need to add the variances to get the resulting REM variance

$$\mathrm{Var}\left(E'(\vec{\theta})\right) = \mathrm{Var}\left(E(\vec{\theta})\right) + \mathrm{Var}\left(E(\vec{\theta}_{ref})\right). \tag{S1}$$

This type of trade-off between the accuracy and the variance is common among mitigation strategies, see for example Cai et al.[15]

To understand the variance that appears in the VQE calculation we can view it as a random process $P(\vec{\theta})$ which generates a random variable X_E corresponding to the minimum energy found by the algorithm, based on some initial set of parameters $\vec{\theta}$. The distribution formed by $P(\vec{\theta})$ is in general not known and is hard to approximate with classical means. The most direct way of learning about $P(\vec{\theta})$ is through repeated sampling of several VQE calculations for the same initial set of parameters. For all calculations presented herein the standard deviations presented are calculated from five repeated runs of the VQE algorithm. The exception being the calculation performed on Chalmers' Särimner device.

The Särimner calculation was not performed as a VQE calculation, but it was instead implemented as an exhaustive sweep of the variational parameter. Thus, the errors in the H_2 calculation are estimated as the sum of weighted Bernoulli samples, where each measurement of a Pauli string, P_i, can be seen as sampling a random variable $X_i \in [-1, 1]$, giving either the +1 eigenstate with probability p_i, or the -1 eigenstate with probability $q_i = 1 - p_i$. The variance of the mean of such sampling is $\mathrm{Var}(\bar{X}_i) = \frac{p_i q_i}{n_i}$ where n_i is the number of samples (for all our measurements $n_i = 5000$). We further assume the measurement of each Pauli string to be independent, which allows for the variances of commuting Pauli strings to be summed into an effective variance $\mathrm{Var}(\bar{X}_s) = \sum_r \mathrm{Var}(\bar{X}_r)$, where r is the index over commuting Pauli strings and s is the index over the resulting non-commuting sets of Pauli strings. The resulting non-commuting effective variances are weighted with the square of their respective Hamiltonian coefficients and summed into the total variance, $\mathrm{Var}(\bar{X}_E) = \sum_s h_s^2 \mathrm{Var}(\bar{X}_s)$. Here the square of the coefficient comes from the definition of variance, $\mathrm{Var}(X) = \mathrm{E}[X^2] - \mathrm{E}[X]^2$. Finally, the standard deviation is obtained by calculating $\sqrt{\mathrm{Var}(\bar{X}_E)}$.

References

(1) Koch, J.; Yu, T. M.; Gambetta, J.; Houck, A. A.; Schuster, D. I.; Majer, J.; Blais, A.; Devoret, M. H.; Girvin, S. M.; Schoelkopf, R. J. Charge-Insensitive Qubit Design Derived from the Cooper Pair Box. *Phys Rev A* **2007**, *76* (4), 042319.

(2) McKay, D. C.; Filipp, S.; Mezzacapo, A.; Magesan, E.; Chow, J. M.; Gambetta, J. M. Universal Gate for Fixed-Frequency Qubits via a Tunable Bus. *Phys Rev Appl* **2016**, *6* (6), 064007.

(3) Bengtsson, A.; Vikstål, P.; Warren, C.; Svensson, M.; Gu, X.; Kockum, A. F.; Krantz, P.; KriŽan, C.; Shiri, D.; Svensson, I. M.; Tancredi, G.; Johansson, G.; Delsing, P.; Ferrini, G.; Bylander, J. Improved Success Probability with Greater Circuit Depth for the Quantum Approximate Optimization Algorithm. *Phys Rev Appl* **2020**, *14* (3), 1.

(4) D'Agostini, G. A Multidimensional Unfolding Method Based on Bayes' Theorem. *Nucl Instrum Methods Phys Res A* **1995**, *362* (2–3), 487–498.

(5) Nocedal, J.; Wright, S. J. *Numerical Optimization. Springer. ISBN 978-0-387-30303-1*; Springer US, 2006.

(6) Sun, Q.; Berkelbach, T. C.; Blunt, N. S.; Booth, G. H.; Guo, S.; Li, Z.; Liu, J.; McClain, J. D.; Sayfutyarova, E. R.; Sharma, S.; Wouters, S.; Chan, G. K. L. PySCF: The Python-Based Simulations of Chemistry Framework. *Wiley Interdiscip Rev Comput Mol Sci* **2018**, *8* (1).

(7) McArdle, S.; Endo, S.; Aspuru-Guzik, A.; Benjamin, S. C.; Yuan, X. Quantum Computational Chemistry. *Rev Mod Phys* **2020**, *92* (1), 015003.

(8) ANIS, M. D. S.; Abraham, H.; AduOffei; Agarwal, R.; Agliardi, G.; Aharoni, M.; Akhalwaya, I. Y.; Aleksandrowicz, G.; Alexander, T.; Amy, M.; Anagolum, S.; Arbel, E.; Asfaw, A.; Athalye, A.; Avkhadiev, A.; Azaustre, C.; BHOLE, P.; Banerjee, A.; Banerjee, S.; Bang, W.; Bansal, A.; Barkoutsos, P.; Barnawal, A.; Barron, G.; Barron, G. S.; Bello, L.; Ben-Haim, Y.; Bevenius, D.; Bhatnagar, D.; Bhobe, A.; Bianchini, P.; Bishop, L. S.; Blank, C.; Bolos, S.; Bopardikar, S.; Bosch, S.; Brandhofer, S.; Brandon; Bravyi, S.; Bronn, N.; Bryce-Fuller; Bucher, D.; Burov, A.; Cabrera, F.; Calpin, P.; Capelluto, L.; Carballo, J.; Carrascal, G.; Carriker, A.; Carvalho, I.; Chen, A.; Chen, C.-F.; Chen, E.; Chen, J. (Chris); Chen, R.; Chevallier, F.; Chinda, K.; Cholarajan, R.; Chow, J. M.; Churchill, S.; Claus, C.; Clauss, C.; Clothier, C.; Cocking, R.; Cocuzzo, R.; Connor, J.; Correa, F.; Cross, A. J.; Cross, A. W.; Cross, S.; Cruz-Benito, J.; Culver, C.; Córcoles-Gonzales, A. D.; D, N.; Dague, S.; Dandachi, T. el; Dangwal, A. N.; Daniel, J.; Daniels, M.; Dartiailh, M.; Davila, A. R.; Debouni, F.; Dekusar, A.; Deshmukh, A.; Deshpande, M.; Ding, D.; Doi, J.; Dow, E. M.; Drechsler, E.; Dumitrescu, E.; Dumon, K.; Duran, I.; EL-Safty, K.; Eastman, E.; Eberle, G.; Ebrahimi, A.; Eendebak, P.; Egger, D.; Emilio; Espiricueta, A.; Everitt, M.; Facoetti, D.; Farida; Fernández, P. M.; Ferracin, S.; Ferrari, D.; Ferrera, A. H.; Fouilland, R.; Frisch, A.; Fuhrer, A.; Fuller, B.; GEORGE, M.; Gacon, J.; Gago, B. G.; Gambella, C.; Gambetta, J. M.; Gammanpila, A.; Garcia, L.; Garg, T.; Garion, S.; Gates, T.; Gil, L.; Gilliam, A.; Giridharan, A.; Gomez-Mosquera, J.; Gonzalo; de la Puente González, S.; Gorzinski, J.; Gould, I.; Greenberg, D.; Grinko, D.; Guan, W.; Gunnels, J. A.; Gupta, H.; Gupta, N.; Günther, J. M.; Haglund, M.; Haide, I.; Hamamura, I.; Hamido, O. C.; Harkins, F.; Hasan, A.; Havlicek, V.; Hellmers, J.; Herok, Ł.; Hillmich, S.; Horii, H.; Howington, C.; Hu, S.; Hu, W.; Huang, J.; Huisman, R.; Imai, H.; Imamichi, T.; Ishizaki, K.; Ishwor; Iten, R.; Itoko, T.; Ivrii, A.; Javadi, A.; Javadi-Abhari, A.; Javed, W.; Jianhua, Q.; Jivrajani, M.; Johns, K.; Johnstun, S.; Jonathan-Shoemaker; JosDenmark; JoshDumo; Judge, J.; Kachmann, T.; Kale, A.; Kanazawa, N.; Kane, J.;

Kang-Bae; Kapila, A.; Karazeev, A.; Kassebaum, P.; Kelso, J.; Kelso, S.; Khanderao, V.; King, S.; Kobayashi, Y.; Kovi11Day; Kovyrshin, A.; Krishnakumar, R.; Krishnan, V.; Krsulich, K.; Kumkar, P.; Kus, G.; LaRose, R.; Lacal, E.; Lambert, R.; Landa, H.; Lapeyre, J.; Latone, J.; Lawrence, S.; Lee, C.; Li, G.; Lishman, J.; Liu, D.; Liu, P.; Maeng, Y.; Maheshkar, S.; Majmudar, K.; Malyshev, A.; Mandouh, M. el; Manela, J.; Manjula; Marecek, J.; Marques, M.; Marwaha, K.; Maslov, D.; PawełMaszota; Mathews, D.; Matsuo, A.; Mazhandu, F.; McClure, D.; McElaney, M.; McGarry, C.; McKay, D.; McPherson, D.; Meesala, S.; Meirom, D.; Mendell, C.; Metcalfe, T.; Mevissen, M.; Meyer, A.; Mezzacapo, A.; Midha, R.; Miller, D.; Minev, Z.; Mitchell, A.; Moll, N.; Montanez, A.; Monteiro, G.; Mooring, M. D.; Morales, R.; Moran, N.; Morcuende, D.; Mostafa, S.; Motta, M.; Moyard, R.; Murali, P.; Müggenburg, J.; Nadlinger, D.; Nakanishi, K.; Nannicini, G.; Nation, P.; Navarro, E.; Naveh, Y.; Neagle, S. W.; Neuweiler, P.; Ngoueya, A.; Nicander, J.; Nick-Singstock; Niroula, P.; Norlen, H.; NuoWenLei; O'Riordan, L. J.; Ogunbayo, O.; Ollitrault, P.; Onodera, T.; Otaolea, R.; Oud, S.; Padilha, D.; Paik, H.; Pal, S.; Pang, Y.; Panigrahi, A.; Pascuzzi, V. R.; Perriello, S.; Peterson, E.; Phan, A.; Piro, F.; Pistoia, M.; Piveteau, C.; Plewa, J.; Pocreau, P.; Pozas-Kerstjens, A.; RafałPracht; Prokop, M.; Prutyanov, V.; Puri, S.; Puzzuoli, D.; Pérez, J.; Quant02; Quintiii; R, I.; Rahman, R. I.; Raja, A.; Rajeev, R.; Ramagiri, N.; Rao, A.; Raymond, R.; Reardon-Smith, O.; Redondo, R. M.-C.; Reuter, M.; Rice, J.; Riedemann, M.; Rietesh; Risinger, D.; Rocca, M. la; Rodr\'\iguez, D. M.; RohithKarur; Rosand, B.; Rossmannek, M.; Ryu, M.; SAPV, T.; Sa, N. R. C.; Saha, A.; Saki, A. A.-; Sanand, S.; Sandberg, M.; Sandesara, H.; Sapra, R.; Sargsyan, H.; Sarkar, A.; Sathaye, N.; Schmitt, B.; Schnabel, C.; Schoenfeld, Z.; Scholten, T. L.; Schoute, E.; Schulterbrandt, M.; Schwarm, J.; Seaward, J.; Sergi; Sertage, I. F.; Setia, K.; Shah, F.; Shammah, N.; Sharma, R.; Shi, Y.; Shoemaker, J.; Silva, A.; Simonetto, A.; Singh, D.; Singh, P.; Singkanipa, P.; Siraichi, Y.; Siri; Sistos, J.; Sitdikov, I.; Sivarajah, S.; Sletfjerding, M. B.; Smolin, J. A.; Soeken, M.; Sokolov, I. O.; Sokolov, I.; Soloviev, V. P.; SooluThomas; Starfish; Steenken, D.; Stypulkoski, M.; Suau, A.; Sun, S.; Sung, K. J.; Suwama, M.; Słowik, O.; Takahashi, H.; Takawale, T.; Tavernelli, I.; Taylor, C.; Taylour, P.; Thomas, S.; Tillet, M.; Tod, M.; Tomasik, M.; de la Torre, E.; Toural, J. L. S.; Trabing, K.; Treinish, M.; Trenev, D.; TrishaPe; Truger, F.; Tsilimigkounakis, G.; Tulsi, D.; Turner, W.; Vaknin, Y.; Valcarce, C. R.; Varchon, F.; Vartak, A.; Vazquez, A. C.; Vijaywargiya, P.; Villar, V.; Vishnu, B.; Vogt-Lee, D.; Vuillot, C.; Weaver, J.; Weidenfeller, J.; Wieczorek, R.; Wildstrom, J. A.; Wilson, J.; Winston, E.; WinterSoldier; Woehr, J. J.; Woerner, S.; Woo, R.; Wood, C. J.; Wood, R.; Wood, S.; Wootton, J.; Wright, M.; Xing, L.; Yang, B.; Yeralin, D.; Yonekura, R.; Yonge-Mallo, D.; Young, R.; Yu, J.; Yu, L.; Zachow, C.; Zdanski, L.; Zhang, H.; Zoufal, C.; aeddins-ibm; alexzhang13; b63; bartek-bartlomiej; bcamorrison; brandhsn; catornow; charmerDark; deeplokhande; dekel.meirom; dime10; dlasecki; ehchen; fanizzamarco; fs1132429; gadial; galeinston; georgezhou20; georgios-ts; gruu; hhorii; hykavitha; itoko; jessica-angel7; jliu45; jscott2; klinvill; krutik2966; ma5x; michelle4654; msuwama; ntgiwsvp; ordmoj; sagar pahwa; pritamsinha2304; ryancocuzzo; saswati-qiskit; septembrr; sethmerkel; shaashwat; sternparky; strickroman; tigerjack; tsura-crisaldo; vadebayo49; welien; willhbang; wmurphy-collabstar; yang.luh; Čepulkovskis, M. Qiskit: An Open-Source Framework for Quantum Computing. 2021.

(9) Kandala, A.; Mezzacapo, A.; Temme, K.; Takita, M.; Brink, M.; Chow, J. M.; Gambetta, J. M. Hardware-Efficient Variational Quantum Eigensolver for Small Molecules and Quantum Magnets. *Nature* **2017**, *549* (7671), 242–246.

(10) Newville, M.; Otten, R.; Nelson, A.; Ingargiola, A.; Stensitzki, T.; Allan, D.; Fox, A.; Carter, F.; Michał; Osborn, R.; Pustakhod, D.; lneuhaus; Weigand, S.; Glenn; Deil, C.; Mark; Hansen, A. L. R.; Pasquevich, G.; Foks, L.; Zobrist, N.; Frost, O.; Beelen, A.; Stuermer; azelcer; Hannum, A.; Polloreno, A.; Nielsen, J. H.; Caldwell, S.; Almarza, A.; Persaud, A. Lmfit/Lmfit-Py: 1.0.3. **2021**.

(11) Johansson, J. R.; Nation, P. D.; Nori, F. QuTiP: An Open-Source Python Framework for the Dynamics of Open Quantum Systems. *Comput Phys Commun* **2012**, *183* (8), 1760–1772.

(12) *IBM Quantum*. https://quantum-computing.ibm.com/ (accessed 2021-12-11).

(13) Georgopoulos, K.; Emary, C.; Zuliani, P. Modelling and Simulating the Noisy Behaviour of Near-Term Quantum Computers. **2021**, 1–13.

(14) Vovrosh, J.; Khosla, K. E.; Greenaway, S.; Self, C.; Kim, M.; Knolle, J. Simple Mitigation of Global Depolarizing Errors in Quantum Simulations. **2021**, 1–10.

(15) Cai, Z.; Babbush, R.; Benjamin, S. C.; Endo, S.; Huggins, W. J.; Li, Y.; McClean, J. R.; O'Brien, T. E. Quantum Error Mitigation. *arXiv:2210.00921 [quant-ph]. arXiv.org ePrint archive*. October 3, 2022. http://arxiv.org/abs/2210.00921 (accessed 2023-01-10).
